# Recurrent genetic variants and prioritization of variants of uncertain clinical significance associated with hereditary breast and ovarian cancer in families from the Region of Murcia

**DOI:** 10.1515/almed-2023-0103

**Published:** 2023-09-22

**Authors:** Laura Rosado-Jiménez, Younes Mestre-Terkemani, Ángeles García-Aliaga, Miguel Marín-Vera, José Antonio Macías-Cerrolaza, María Desamparados Sarabia-Meseguer, María Rosario García-Hernández, Marta Zafra-Poves, Pilar Sánchez-Henarejos, Francisco Ayala de la Peña, José Luis Alonso-Romero, José Antonio Noguera-Velasco, Francisco Ruiz-Espejo

**Affiliations:** Genomics Laboratory, Department Service of Clinical Laboratory Analysis, Clinical University Hospital Virgen de la Arrixaca, Murcia, Spain; Service of Medical Oncology, Virgen de la Arrixaca University Hospital, Murcia, Spain; Department of Medical Oncology, Clinical University Hospital Morales Meseguer, Murcia, Spain

**Keywords:** founder effect, mutational spectrum, prioritization, hereditary breast and ovarian cancer, variants of uncertain clinical significance, recurrent pathogenic variants

## Abstract

**Objectives:**

Hereditary breast and ovarian cancer (HBOC) follows an autosomal dominant inheritance pattern of cancer susceptibility genes. The risk of developing this disease is primarily associated with germline mutations in the *BRCA1* and *BRCA2* genes. The advent of massive genetic sequencing technologies has expanded the mutational spectrum of this hereditary syndrome, thereby increasing the number of variants of uncertain clinical significance (VUS) detected by genetic testing.

**Methods:**

A prevalence study of HBOC was performed within 2,928 families from the Region of Murcia, in southeastern Spain. Genetic testing enabled the identification of recurrent pathogenic variants and founder mutations, which were mainly related to the *BRCA1* and *BRCA2* genes. VUS testing was performed using a prioritization algorithm designed by our working group.

**Results:**

Variants c.68_69del, c.212+1G>A, and c.5123C>A were detected in 30 % of *BRCA1* carriers, whereas exon 2 deletion concurrent with c.3264dupT, c.3455T>G and c.9117G>A variants were found in 30 % of *BRCA2* carriers. A total of 16 VUS (15 %) were prioritized.

**Conclusions:**

The genotype-phenotype correlation observed in our study is consistent with the scientific literature. Furthermore, the founder effect of c.1918C>T (*BRCA1*) and c.8251_8254del (*ATM*) was verified in the Murcian population, whereas exon 2 deletion (*BRCA2*) was proven to be a Spanish founder mutation. Our algorithm enabled us to prioritize potentially pathogenic VUS that required further testing to determine their clinical significance and potential role in HBOC.

## Introduction

The latest report of the International Agency for Research on Cancer (IARC) reveals a change of tendency in the epidemiology of cancer. Thus, breast cancer was the most commonly diagnosed cancer worldwide (11.7 %) in 2020 [1], globally accounting for 24.5 % of new cases of cancer in women. This type of cancer is the leading cause of cancer-related deaths in women worldwide (15.5 %) and the second leading cause of death in Spain (14.6 %) [2].

On a global scale, ovarian cancer is the third most recurrent gynecological tumor, accounting for 3.4 % of the global incidence in women and 4.7 % of cancer-related deaths in the female population [2].

About 7 % of breast tumors and 11–15 % of epithelial ovarian tumors are hereditary, being associated with germline mutations in cancer susceptibility genes, primarily *BRCA1* and *BRCA2* [3]. Advances in hereditary breast and ovarian cancer (HBOC) genetic testing have made it possible to identify novel genes with variable genetic penetrance and demonstrated clinical actionability for HBOC. In addition, other genes whose clinical validity is still under investigation have also been discovered [4, 5].

The emergence of next generation sequencing technologies, added to the recent incorporation of multigene panel testing, has been a breakthrough in the molecular diagnosis of HBOC. Nonetheless, the interpretation of results poses a challenge to laboratory medicine specialists due to the exponential increase of new genes analyzed. Uncertainty about the clinical significance of variants leads to the reporting of non-informative results, complicating genetic counseling [6]. For this reason, gene panels should only include clinically actionable genes, in addition to a regular review of the variant of uncertain significance (VUS) detected and follow-up of VUS carriers that should be performed, pending new scientific evidence that enables their clinical classification.

Likewise, it is crucial that prioritization criteria are developed on the basis of the clinical actionability of genes, *in silico* target predictions, and the scientific evidence available. Variant prioritization makes it possible to identify potentially pathogenic VUS that may require further testing to assess their biological impact and potential role in HBOC, as compared to other VUS [7].

On another note, the identification of recurrent founder pathogenic variants associated with HBOC helps define the mutational profile of a specific population. In this way, tailored gene panels can be designed for specific population groups, thereby facilitating specialized genetic counseling.

Rebbeck et al. conducted a worldwide study on the mutational spectrum and prevalence of susceptibility genes *BRCA1/2* by geographical origin and race/ethnicity (see Supplementary Material, Tables 1 and 2). Although the studied population reveals some genetic diversity, evidence demonstrates that the most common genetic variants in all world regions were the so-called founder mutations in Ashkenazi Jewish: c.68_69del (BIC: 185delAG), c.5266dup (BIC: 5382insC) in *BRCA1*, and c.5946del (BIC: 6174delT) in *BRCA2* [8].

In Spain, the mutational spectrum of *BRCA1/2* shows considerable variations across the different Spanish population groups. Of note, there is a spectrum of recurrent pathogenic variants in Spanish HBOC families. Concerning *BRCA1*, c.211A>G is the most prevalent genetic variant in the Spanish population (especially in northwest Spain) [9], followed by c.68_69del and c.5123C>A. The historical presence of Jews (Sephardic) in the Iberian Peninsula explains that these two variants are highly prevalent and widely distributed across the national territory [10, 11]. The following most prevalent pathogenic variants in the Spanish population are c.3770_3771del and c.3331_3334del. In regard to the c.3331_3334del variant, haplotype analysis in Hispanic carriers suggests that this ancestral mutation originated in the Iberian Peninsula and spread to Latin America during the colonization [12]. As for *BRCA2*, the c.2808_2811del and c.6275_6276del variants are widely distributed across the Spanish population. The diversity of c.2808_2811del haplotypes suggests multiple separate origins. Nevertheless, haplotype analysis of the c.6275_6276del variant reported in a variety of populations worldwide indicates that it may have originated in northern Europe [13]. In contrast, the genetic variants c.3264dup, c.9026_9030del, and c.9018C>A are more recurrent in the Mediterranean basin [14].

## Materials and methods

### Selection of high-risk families

A total of 2,928 families from the Region of Murcia were selected between April 2007 and April 2022. These families met the high-risk criteria for the indication of HBOC genetic testing established by the Spanish Society of Medical Oncology (SEOM) and the Genetic Counseling Unit of the Region of Murcia (Supplementary Material, Table 3) [3, 15].

### HBOC genetic testing in the Region of Murcia

**Figure 1:**
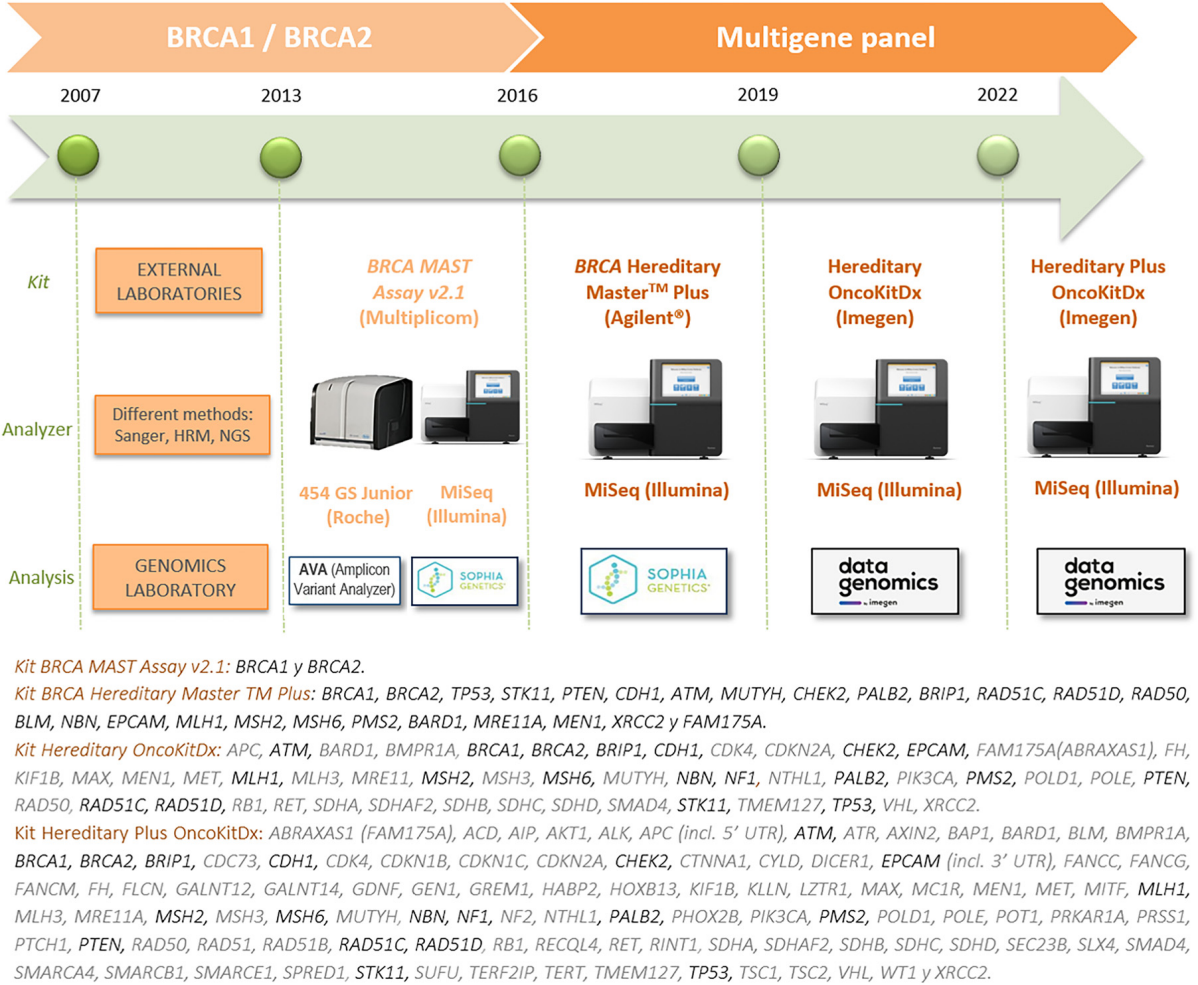
Timeline of molecular diagnosis for HBOC. The genes selected for the clinical bioinformatic study are represented in bold (Hereditary OncokitDx kit and Hereditary Plus OncokitDx kit) according to NCCN v.3.2019 recommendations.

Figure 1 shows the genetic testing method used for the diagnosis of HBOC. This figure displays a timeline of the evolution of HBOC molecular diagnostics, which were initially based on testing for *BRCA1* and *BRCA2* and evolved to massive sequencing of a panel that includes clinically actionable genes for breast and ovarian cancer (*BRCA1*, *BRCA2*, *TP53*, *PTEN*, *CDH1*, *STK11*, *PALB2*, *ATM*, *CHEK2*, *NBN*, *NF1*, *BRIP1*, *RAD51C*, *RAD51D*, *MSH2*, *MLH1*, *MSH6*, *PMS2*, *EPCAM*), recommended by the *National Comprehensive Cancer Network* (NCCN v.3.2019) [16].

Following genetic testing, all the genetic variants detected were classified according to the consensus criteria of the American College of Medical Genetics and Genomics and to the databases and literature sources available [17].

### Mutational spectrum of HBOC in the Region of Murcia

Analysis of the mutational profile of HBOC permitted the identification of recurrent clinically relevant variants in 2,928 families from the Region of Murcia.

We selected highly prevalent variants classified as pathogenic by the ACMG. The minimal value for a pathogenic variant to be classified as recurrent was set at 10 carrier families per variant, which accounted for >5 % of the population of *BRCA1*/*BRCA2* carriers.

### Genotype-phenotype correlation study

A genotype-phenotype correlation study was performed in families with carriers of a recurrent pathogenic variant, considering the age at tumor diagnosis, geographical origin, type of cancer, and histopathological and immunohistochemical features. Prevalence and phenotypic expression were compared with those reported in other Spanish and international studies on HBOC.

### Statistical analysis

Descriptive and inferential statistical analyses were performed using the SPSS v.27 software package.

– Quantitative variables were presented as central tendency and dispersion measures. Qualitative variables were expressed as absolute and relative frequencies.
– Differences in qualitative variables were assessed using Pearson’s chi square test. Differences were considered to be significant when the p-value was <0.05.

### Pathogenic variants with founder effect in the Region of Murcia

Haplotype analysis of genetic variants with a potential founder effect in the Region of Murcia was performed by capillary electrophoresis of labeled microsatellites using the Type-it Microsatellite PCR kit (Qiagen), under the conditions described in the Supplementary Material, Table 4 and Figure 1.

**Figure 2:**
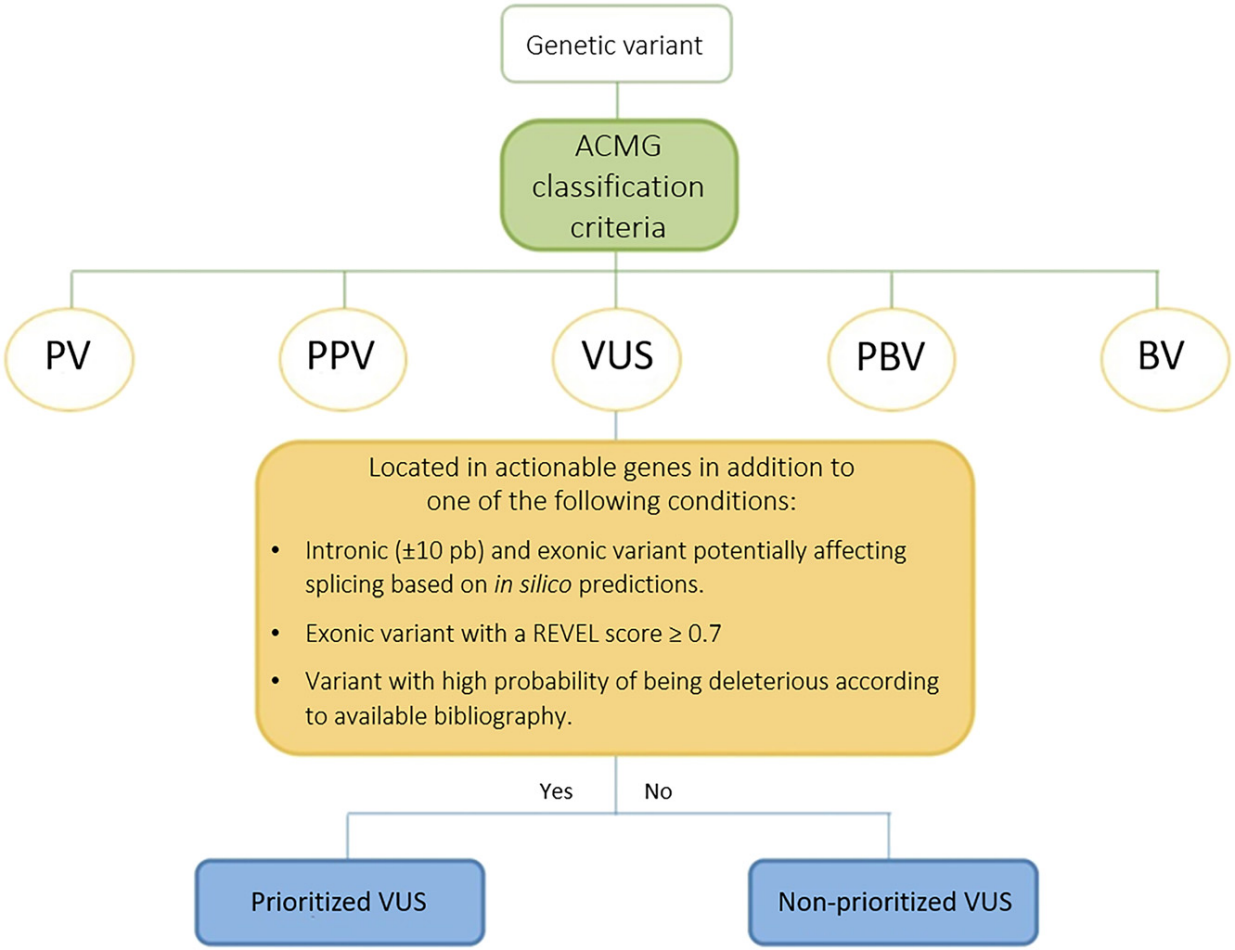
Algorithm used for VUS prioritization. PV, pathogenic variant; PPV, probably pathogenic variant; VUS, variant of uncertain clinical significance; PBV, probably benign variant; BV, benign variant; bp, base pairs.

**Figure 3:**
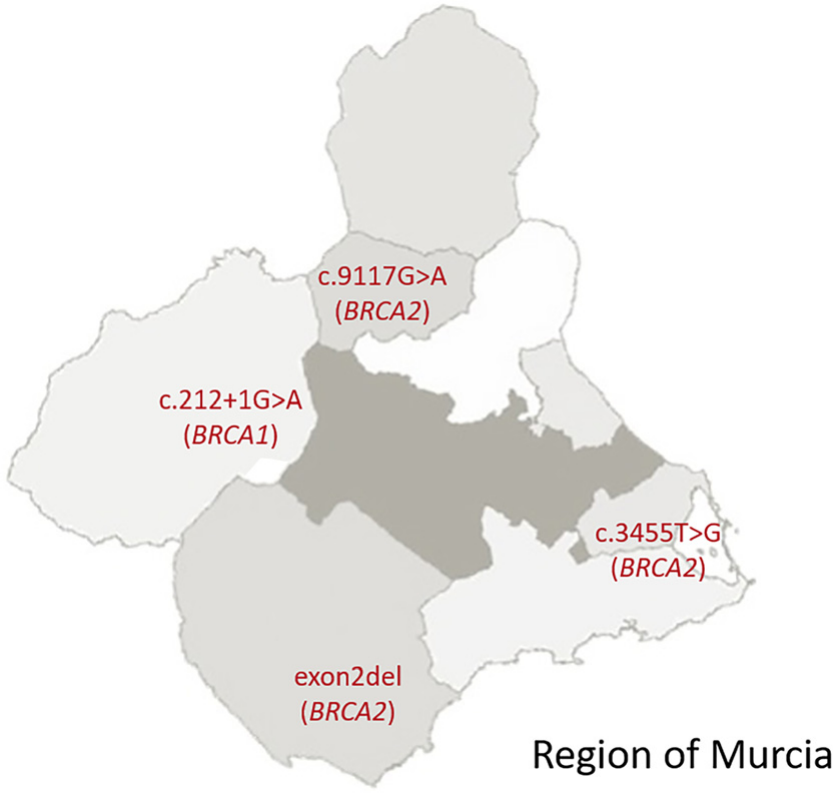
Geographic distribution of the pathogenic variants frequently detected in families from the Region of Murcia.

The microsatellites selected for haplotype analysis of variants c.1918C>T in *BRCA1*, exon 2 deletion in *BRCA2* and c.8251_8254del in ATM, and the position of these markers in chromosomes are shown in the Supplementary Material Tables 5–7 and Figures 2–4.

In parallel, the technique designed for each variant was used in 20 control samples from a gene bank of the Genomics Laboratory of Clinical University Hospital Virgen de la Arrixaca (patients from different areas of the Region of Murcia without any apparent consanguineal kinship, not meeting SEOM criteria). The aim was to demonstrate that the common haplotype was not found in the control population and estimate when these mutations occurred.

The number of generations of founder mutations was estimated using the equation developed by Machado et al. [18]. G=logδ/log(1 − θ). δ indicates the linkage disequilibrium measure between the mutation and each of the closest recombinant microsatellite markers. It is calculated from the frequency of the ancestral allele (Pd) and the frequency of that microsatellite control allele on control chromosomes (Pn) using the formula δ=(Pd − Pn)/(1 − Pn). The symbol θ represents the recombination fraction between a marker and the gene, calculated from the distances between the markers and the gene, obtained from the *Ensembl* database [19].

### Study of variants of uncertain clinical significance

The VUS detected in HBOC genetic testing were reviewed using different databases and bioinformatic tools. A research was also performed for new scientific evidence that might lead to VUS reclassification.

Then, the VUS reclassified as such were processed using the prioritization algorithm designed by our working group (Figure 2).

The use of prioritization criteria helped to identify VUS found in clinically actionable genes recommended by the NCCN [16] that met at least one of the following criteria:

– Intronic variants located at the exon-intron border (±10 pb) or exonic variants predicted to cause a splicing aberration by *in silico* splice-site tools (SpliceSiteFinder, MaxEntScan, NNSplice and *GeneSplicer*), which were included in Varsome and Alamut [20].
– Exonic variants with a score ≥0.7 on REVEL score (Rare Exome Variant Ensemble Learner). This method predicts the pathogenicity of missense variants using different bioinformatic programs: MutPred, FATHMM, VEST, PolyPhen, SIFT, PROVEAN, MutationAssesor, Mutation Taster, LRT, GERP, SiPhy, phyloP, and phastCons*.*
– The threshold score that discriminated benign from pathogenic variants was 0.7, showing a sensitivity of 0.5786 and a specificity of 0.9556 [21].
– Variants with high probability of being deleterious according to available bibliography on PubMed and Varsome [22, 23].

Therefore, prioritization of VUS makes it possible to optimize further testing (analysis of familial co-segregation, case-control studies, or functional clinical trials) on the variants that are most likely to be deleterious, in order to determine their clinical significance and potential role in HBOC.

## Results and discussion

### Mutational spectrum of HBOC in the Region of Murcia

The analysis of the mutational spectrum of HBOC in the Region of Murcia displays the existing genetic variability in the local population. The results of this study warrant the use of a specific panel of clinically actionable genes that confer a higher risk of developing this hereditary cancer syndrome.

The prevalence study carried out in 2,928 HBOC families contributed to the identification of recurrent pathogenic variants and founder mutations in the Region of Murcia (Table 1). This study reveals that the genes harboring higher rates of clinically relevant mutations were the main cancer susceptibility genes, *BRCA1/2*, followed by a relevant percentage of high to moderate penetrance genes (*ATM, CHEK2, PALB2, BRIP1, and TP53*). These results are consistent with the literature [24].

**Table 1:** Recurrent and founder *BRCA1/2* variants in the Region of Murcia.

Gene	Exon/intron	HGVS	refSNP	Type of variant	ClinVar	No. of families	Dominant phenotype
*BRCA1*	2	c.68_69del	rs80357914	Frameshift	PV	23	BC: ID TN
	IN4-5	c.212+1G>A	rs80358042	Intronic	PV	16	BC: ID TN
	10	c.1918C>T	rs886039981	Nonsense	PV	9	BC: ID TN
	17	c.5123C>A	rs28897696	Missense	PV	12	BC: ID TN
*BRCA2*	2	exon2del	–	LGR	PV	12	BC: ID LA
	11	c.3264dup	rs80359380	Frameshift	PV	10	BC: ID LA
	11	c.3455T>G	rs80358593	Nonsense	PV	12	BC: ID LA
	23	c.9117G>A	rs28897756	Synonymous	PV	16	BC: ID LA
*ATM*	56	c.8251_8254del	rs786202120	Frameshift	PV	8	BC: ID LA

BC, breast cancer; DI, invasive ductal; HGVS, Human Genome Variation Society; LA/B, luminal A/B; LGR, large genomic rearrangements; TN, triple negative; PV, pathogenic variant.

This study revealed that variants c.68_69del, c.212+1G>A, and c.5123C>A were detected in 30 % of *BRCA1+* patients. Additionally, exon 2 deletion, along with c.3264dup, c.3455T>G and c.9117G>A variants were found in 30 % of *BRCA2+* cases [25].

### Phenotype of BRCA1/2+ families

The genotype–phenotype correlation demonstrated statistically significant differences between the recurrent pathogenic variants *BRCA1* and *BRCA2* detected in carrier families from the Region of Murcia. The dominant histological type of breast cancer in *BRCA1+* patients was triple-negative invasive ductal carcinoma. In contrast, a more heterogeneous phenotype was observed in *BRCA2+* patients diagnosed with breast cancer. Nonetheless, the predominant phenotype has been invasive ductal carcinoma with estrogen receptor-positive and HER2-negative (luminal A). Most of *BRCA1+* tumors turned to be infiltrative ductal carcinomas, however luminal A estrogen receptor-positive and HER2-negative was the most common phenotype. Therefore, the histopathological and immunophenotypic characteristics of *BRCA1/2+* breast tumors observed in this study are consistent with the literature [26]. The median age at diagnosis of *BRCA1/BRCA2+* breast cancer exceeded 40 years.

Likewise, *BRCA1* c.68_69del and c.5123C>A variants were associated with a higher number of cases of bilateral breast cancer. In addition, the prevalence of male breast cancer was higher in carriers of *BRCA2* variants. The histopathological and immunohistochemical profile of synchronous and metachronous bilateral breast carcinomas showed a concordance of 65 %, which is lower than such described in previous studies [27]. This finding suggests that the phenotypic expression of the primary tumor was relatively predictive of the expression status of the secondary tumor. Nevertheless, since the phenotype of the primary tumor is not always predictive, it is necessary to determine the histopathological and immunohistochemical profile of each tumor in order to ensure appropriate diagnosis and therapeutic decision-making.

As expected, more significant differences were found in the phenotypic characteristics of breast carcinomas, as compared to ovarian carcinomas, which showed a predominant phenotype. Consistently with the literature [26], virtually all *BRCA* carriers with ovarian carcinoma received a diagnosis of high-grade serous ovarian carcinoma [26]. Likewise, the median age at diagnosis of ovarian cancer exceeded 50 years.

### Geographic origin of carrier families

There was heterogeneity in the geographic origin of carriers of c.68_69del (*BRCA1*), c.5123C>A (*BRCA1*), and c.3264dup (*BRCA2*), as these variants were widely distributed across the Region de Murcia.

On the contrary, families with carriers of other recurrent pathogenic variants came from specific geographical areas. Thus, the distribution of exon 2 deletion (*BRCA2*) was restrained to Valle del Guadalentín; c.212+1G>A (*BRCA1*) was more common in the northwest area; c.9117G>A (*BRCA2*) was more frequent in Vega Alta del Segura; and c.3455T>G (*BRCA2*) was mostly found in the southeastern area of the Region of Murcia (Figure 3).

It is worth mentioning that whereas the genetic variants with a heterogeneous distribution across the Region of Murcia are consistent with those widely described in the national territory. Exon 2 deletion (*BRCA2*), c.212+1G>A (*BRCA1*), c.3455T>G (*BRCA2*) and c.9117G>A (*BRCA2*) variants are distinctive of the Region.

### Comparative study of mutational prevalence

Comparative analysis between the Murcian population, and the European and Spanish population revealed that the prevalence of the *BRCA1* mutation was more similar to such reported in previous studies than *BRCA2*. Notably, we observed some genetic affinity with other populations from the Mediterranean basin, due to geographical proximity with our study population.

Regarding large rearrangements, global studies report a higher frequency in *BRCA1*, as compared to *BRCA2*, which is in agreement with previous studies in the Spanish population. Genomic rearrangements in *BRCA1* account for 2.1 % of Spanish families with hereditary breast and ovarian cancer, whereas *BRCA2* explains 1.5 % [28, 29]. It is worth mentioning that the results of our study are inconsistent with the literature, as they reveal a higher prevalence of families carrying genomic rearrangements in *BRCA2* (4.1 % *BRCA1* vs. 7.8 % *BRCA2*), with exon 2 deletion being the most frequent genomic rearrangement in our study population.

### Pathogenic variants with founder effect in the Region of Murcia

Microsatellite instability analysis confirmed the presence of a common haplotype of two founder mutations in the Region of Murcia: the c.1918C>T (*BRCA1*) variant, detected exclusively in our study population [30], and the c.8251_8254del (*ATM*) [31] (see Supplementary Material, Table 8 and Figure 5). Furthermore, the two variants emerged recently, as they are estimated to have occurred 18 and 7 generations ago, respectively [32].

In the same way, exon 2 deletion (*BRCA2*) has been proven to be a Spanish founder mutation, since the presence of a common ancestor with the families studied by Ruiz de Garibay et al. [33] has been demonstrated (see Supplementary Material, Table 9). This mutation was estimated to have occurred 22 generations ago, which means that it is a recent founder mutation [34]. The noticeable founder effect observed in the population of Murcia would explain a significant proportion of the genomic rearrangements detected in *BRCA2*.

### Study of variants of uncertain clinical significance

Regarding hereditary susceptibility genes, the clinical interpretation of genetic variants is crucial for genetic counseling. The exponential increase in the number of VUS detected makes it necessary to implement effective review strategies to perform further testing on the prioritized VUS [35].

In our study population, a total of 115 VUS were detected in clinically actionable genes, being the majority missense mutations. Even though seven VUS were reclassified as benign or likely pathogenic, 93 % of all variants were confirmed to be VUS despite reclassification efforts (Table 2). These results are consistent with previous variant reclassification studies, with a higher number of variants reclassified as benign [36].

**Table 2:** VUS reclassification.

Gene	Exon/intron	HGVS	Type of variant	ClinVar	HGMD	Varsome	ACMG criteria	Reclassification
*BRCA1*	10	c.2634A>G	Synonymous	PBV	ND	BV	BP4 + BP6 + BP7	PBV
		c.2770A>G	Missense	PBV	ND	PBV	PM2, BP3 + BP4	PBV
		c.3759T>A	Synonymous	PBV	ND	PBV	BP4 + BP6 + BP7	PBV
	23	c.5507_5508del	Frameshift	ND	DM	PPV	PVS1, PM2	PPV
*BRCA2*	IN 5	c.475+14C>T	Intronic	PBV	ND	PBV	BP4 + BP6	PBV
	IN 13	c.7008-14del	Intronic	PBV	ND	PBV	BP6	PBV
*ATM*	IN 8	c.1066-6T>G	Intronic	BV	VUS	PBV	BS1 + BS2, BP6, PP5	BV
	IN 45	c.6572+11C>T	Intronic	PBV	ND	PBV	BP4 + BP6	PBV

DM, disease-causing mutation; HGMD, Human Gene Mutation Database; HGVS, Human Genome Variation Society; ND, not described; PPV, probably pathogenic variant; VUS, variant of uncertain significance; PBV, probably benign variant; BV, benign variant.

The algorithm developed by our working group made it possible to prioritize 16 variants, which accounted for 15 % of the VUS detected (Table 3).

**Table 3:** VUS prioritized along with the phenotype associated with carriers and prioritization criteria.

Gene	HGVS	refSNP	Type of variant	No. of cases	Index case	Prioritization criterion	
*ATM*	c.967A>G	rs587781511	Missense	1	BC (47)	Literature	Li et al. [37] George Priya Doss et al. [38] Carranza et al. [39] Fiévet et al. [40]
	c.3402+3A>C	rs786203688	Intronic	1	BC (34)	Splicing	Varsome (0.9998)
	c.4388T>G	rs138327406	Missense	2	OC (34)	REVEL	Pathogenic (0.758)
					BC (49)		
	c.6067G>A	rs11212587	Missense	1	bCM (39)	Literature	Mangone et al. [41] Podralska et al. [42] Thorstenson et al. [43]
*BRCA2*	c.3032C>G	rs80358548	Missense	1	bCM (45, 47)	REVEL	Pathogenic (0.72)
	c.4594G>T	rs80358693	Missense	1	BC (31)	Literature	Ochiai et al. [44]
	c.7559G>T	rs80358982	Missense	1	OC (25)	REVEL	Pathogenic (0.7419)
	c.9275A>G	rs80359195	Missense	3	BC (61)	REVEL	Pathogenic (0.808)
					BC (36)		
					BC (49)		
*CHEK2*	c.320-5T>A	rs121908700	Intronic	2	BC (27)	Splicing	Varsome (0.8037)
					BC (37)		
	c.442A>G	rs876660482	Missense	1	BC (47)	Literature	Delimitsou et al. [45] Apostolou et al. [46]
						REVEL	Pathogenic (0.85)
	c.1008G>A	rs201544715	Synonymous	1	BC (38)	Splicing	Varsome (0.9999)
	c.1427C>T	rs142763740	Missense	1	BC (21)	Literature	Yurgelun et al. [47] Eliade et al. [48] Desrichard et al. [49] Roeb et al. [50]
*MSH2*	c.470G>C	rs765489269	Missense	1	BC (39)	REVEL	Pathogenic (0.763)
	c.1045C>G	rs267607939	Missense	1	BC (42)	REVEL	Pathogenic (0.9929)
*MSH6*	c.3883C>T	ND	Missense	1	OC (39) BC (60)	REVEL	Pathogenic (0.7059)
*NBN* ^a^	c.2071-4A>G	rs746994234	Intronic	1	BC (47)	Splicing	Varsome (0.9987)

^a^A significant limitation of the VUS prioritization study was the unavailability of the biological sample required for *in vitro* testing for the prioritized variant c.2071-4A>G in *NBN,* which may potentially affect the splicing pattern, as predicted by computational programs. BC, breast cancer; bCM, bilateral BC; OC, ovarian cancer; HGVS, Human Genome Variation Society; ND, not described; REVEL, Rare Exome Variant Ensemble Learner.

To conclude, variants were prioritized because of their potential pathogenicity on the basis of the following scientific evidence:

– The c.967A>G variant results in an isoleucine-for-valine substitution at residue 323 of the ATM protein. Although the different bioinformatic tools yield inconsistent predictions, the potential pathogenicity of this variant should be considered, as it has been described in patients with ataxia-telangiectasia and has been classified as deleterious due to its impact on ATM function [37], [38], [39], [40].
– Variant c.6067G>A causes a glycine-for-arginin substitution at residue 2023 of the ATM protein. Most bioinformatic tools identify it as pathogenic since this variant has been previously reported in breast cancer patients. Notwithstanding that it has been predicted as potentially pathogenic, its pathogenicity has not been confirmed by functional or case-control studies [41], [42], [43]. Therefore, further studies are necessary to draw robust conclusions on the deleterious effect of this variant and its potential association with a higher risk of developing cancer.
– The c.4594G>T variant results in the substitution of valine for phenylalanine at codon 1,532 of the BRCA2 protein. Most bioinformatic tools identify this variant documented in breast cancer patients as pathogenic. Experimental studies demonstrate that valine 1,532 plays a major role in the interaction between BRCA2 and RAD51. Therefore, the V1532F variant debilitates BRC4–RAD51 interaction, thereby affecting BRCA2 protein function [44]. According to the functional studies available in the literature, these variants should be considered as potentially pathogenic.
– The c.442A>G variant causes a glycine-for-arginin substitution at residue 148 of the CHEK2 protein. *In silico* tools do not predict a significant impact on splicing, despite the split into three nucleotides at the end of the second encoding gene. In contrast, computational predictions and an *in vivo* functional study carried out in yeast by Delimitsou et al. suggest a potential deleterious effect on this protein [45]. For that reason, its potential pathogenicity should also be considered, since it has been documented in patients with a clinical and/or familial history of inherited breast and ovarian cancer [46].
– The c.1427C>T variant causes a substitution of threonine for methionine at residue 476 of the CHEK2 protein and has been reported in patients with breast and colorectal cancer [47, 48]. This variant is consistently predicted to be deleterious by most bioinformatic tools, as well as in the *in vivo* functional study conducted by Roeb et al. [49, 50]. The alarming phenotype of the index case in our study (a case of breast cancer at 21 years) warrants further research to confirm its pathogenicity through a co-segregation study of this variant.

## Supplementary Material

Supplementary Material
