# Supplementary material for: Recurrent genetic variants and prioritization of variants of uncertain clinical significance associated with hereditary breast and ovarian cancer in families from the Region of Murcia
Source: Adv Lab Med. 2023 Sep 22;4(3):279–87. doi: 10.1515/almed-2023-0103 (PMC10701504; doi:10.1515/almed-2023-0103)

**Supplementary Table 1.** *BRCA1/2* pathogenic variants grouped by geographical region

**
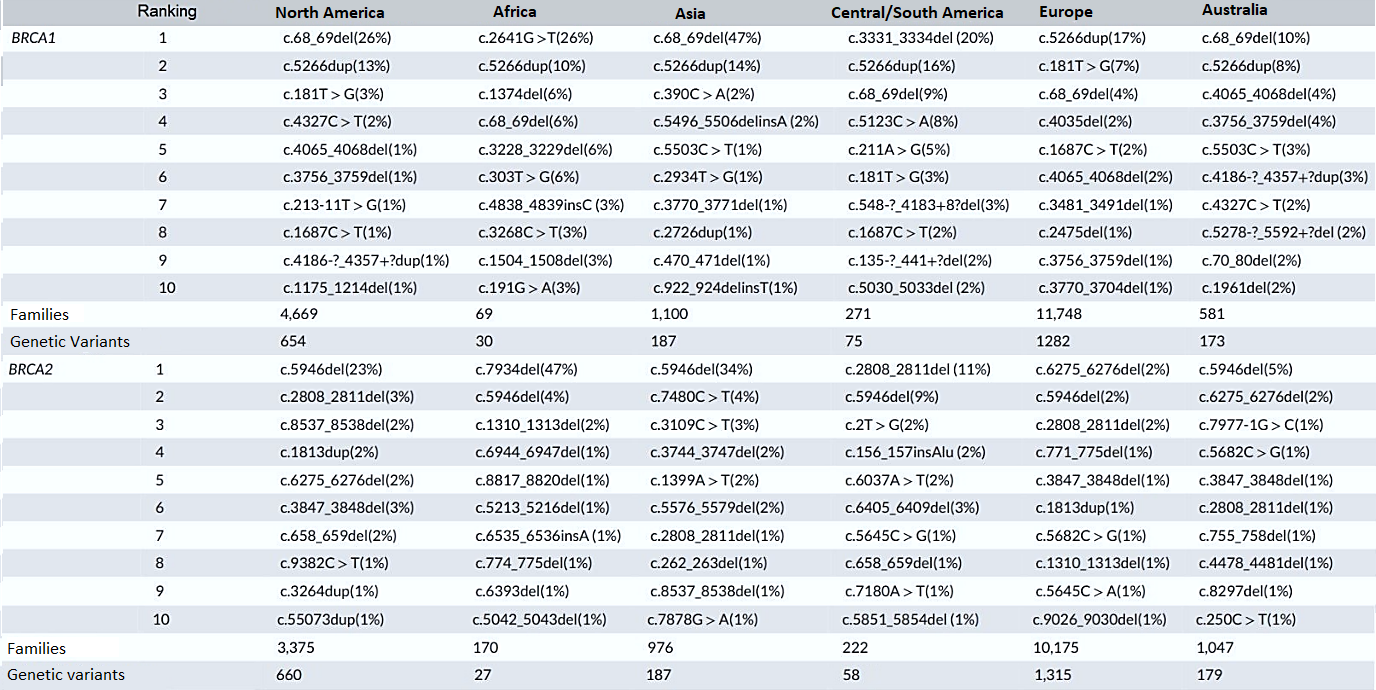
**

**Supplementary Table 2.** *BRCA1/2* pathogenic variants grouped by race/ethnicity

**
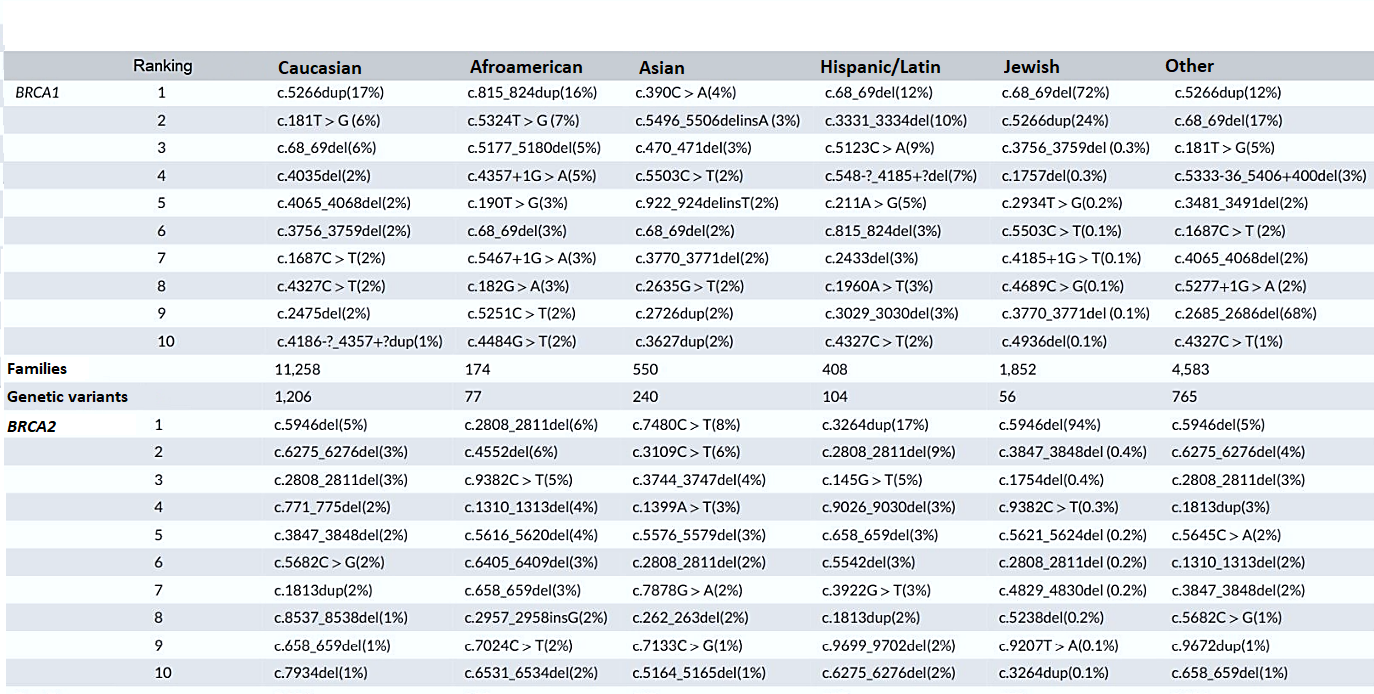

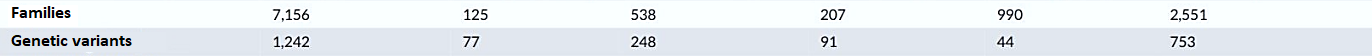
**

**Supplementary Table 3.** Evolution of criteria used in the genetic study for HBOC

1. Criteria for *BRCA1* and *BRCA2* genetic testing established by SEOM [3]

| Regardless of family history |
| --- |
| Women with synchronic or metachronous breast and ovarian cancer |
| Breast cancer at ≤ 35 years (or 40 years in families with less than two women alive at 45 years in each branch of the family) |
| Bilateral breast cancer (primary tumor diagnosed at ≤ 40 years) |
| Triple-negative breast cancer diagnosed at ≤ 50 years. |
| Diagnosis of high-grade epithelial non-mucinous ovarian cancer (or fallopian tube cancer or primary peritoneal cancer). |
| Two or more first-degree relatives presenting any of the following combinations: |
| Bilateral breast cancer and another cancer < 50 years of age |
| Male breast cancer |
| Breast and ovarian cancer |
| Two cases of breast cancer diagnosed < 50 years |
| Three or more direct relatives of the same family branch with breast and/or ovarian cancer |

1. Inclusion criteria established by the Genetic Counseling Unit of the Region of Murcia for HBOC multigene panel testing

| Non-informative *BRCA1* and *BRCA2* genetic testing concurrent with one of the following criteria related to family history of cancer |
| --- |
| Three or more members from two different generations diagnosed with breast or ovarian cancer At least two members of the family diagnosed < 50 years or one < 40 years. |
| Two or more members diagnosed of breast or ovarian cancer < 35 years |
| A case of male breast cancer and another case of breast or ovarian cancer < 50 years. |
| A case of ovarian cancer < 35 years and a case of breast cancer < 50 years in a first-grade relative. |
| Non-informative *BRCA1*  and *BRCA2* genetic testing concurrent with one of the following criteria related to family history of cancer |
| Diagnosis of breast and ovarian cancer in the same patient, provided that one was diagnosed < 50 years. |
| A member of the family diagnosed of breast or ovarian cancer < 25 years |

1. Current SEOM guidelines for the selection of high-risk patients candidate to HBOC genetic testing [15].

| Regardless of family history |
| --- |
| Women with synchronous and metachronous breast and ovarian cancer |
| Breast cancer ≤ 40 years |
| Bilateral breast cancer (primary tumor diagnosed at ≤ 40 years) |
| Triple-negative breast cancer diagnosed at ≤ 60 years. |
| High-grade epithelial non-mucinous ovarian cancer (or Fallopian tube cancer or primary peritoneal cancer). |
| Ancestry with founder mutations |
| BRCA somatic mutation detected in any tumor type with a allele frequency > 30% (if it is known) |
| Metastatic HER2-negative breast cancer patients eligible to consider PARP inhibitor therapy |
| Two or more first-degree relatives presenting any of the following high-risk combinations: |
| Bilateral breast cancer + another breast cancer < 60 years |
| Breast cancer <50 years and prostate and pancreatic cancer <60 years |
| Male breast cancer |
| Breast and ovarian cancer |
| Two cases of breast cancer diagnosed < 50 years |
| Three or more direct relatives with breast cancer (with at least one being premenopausal) and/or ovarian and/or pancreatic cancer or high-grade prostate cancer (Gleason ≥ 7) |

***Supplementary Table 4.*** *Amplification reaction.*GoTaq® Hot Start Polymerase-PCR-Promega kit; F: forward, R: reverse.*

| **PCR amplification** | **Volume (mL)** |
| --- | --- |
| H_2_O | 2 |
| *Master mix** | 6.25 |
| F +R primer (2 µM) | 1.25 |
| *Q-solution** | 2.5 |
| DNA (25 ng/µL) | 0.5 |
| Final volume | 12.5 µL |

**Supplementary Figure 1.** Amplification PCR program on *Veriti HID 96-Well ThermalCycler (Applied Biosystems)*

Following amplification, capillary electrophoresis was carried out on the ABI3130 analyzer by fragment analysis. For such purpose, 1μl of the amplification product was mixed with 12 μl of Hi-Di formamide and 0.5 μl of the LIZ-500 size standard (*Gene Scan-500 LIZ Size Standard-Applied Biosystems- Fisher Scientific)* and was denaturalized for 5 minutes at 95º.

The data obtained after capillary electrophoresis were analyzed using the *Gene Mapper* v 4.0 software package supplied by *Applied Biosystems*


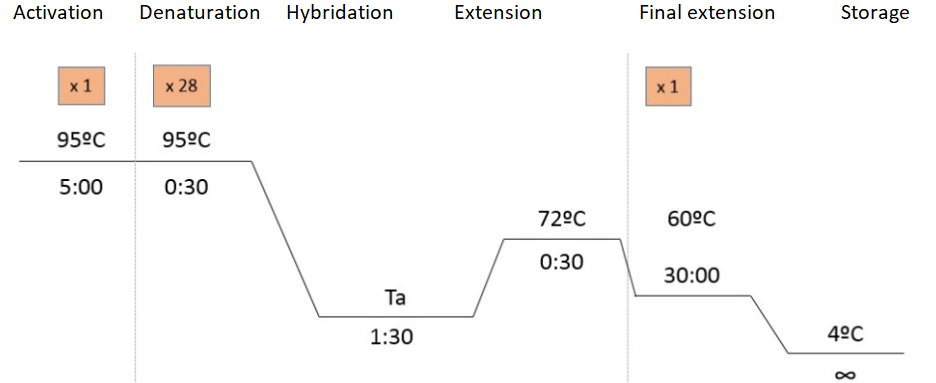


**Supplementary Table 5.** Primer sequences used in the haplotype analysis of the c.1918C>T variant in *BRCA1 (*forward primers were labeled with the FAM fluorophore for use in the analysis of fragments). Temp: *annealing* temperature bp: base pairs

| **MICROSATELLITE MARKERS IN *BRCA1*** | | | | | |
| --- | --- | --- | --- | --- | --- |
| Marker | | *Forward primer* | *Reverse primer* | Temp (ºC) | Size (bp) |
| M1 | D17S855 | [6FAM] GGATGGCCTTTTAGAAAGTGG | ACACAGACTTGTCCTACTGCC | 58 | 139-152 |
| M2 | 17-3858 | [6FAM] CATTTGCTGTTCCCTACCCCACAC | AGAACAATGCAAATTGAAGCAATGA | 58 | 127-134 |
| M3 | D17S1326 | [6FAM] CAGCTGATATTTCACAGGACT | AGAGCAAAACTCCATCTCAAACA | 58 | 89-108 |
| M4 | 17-3930 | [6FAM] ATCTTTGCCTAATCCAGGGTCACAAG | CAAATGGTGCTGGAATAGTTGGA | 57 | 286-298 |

**Supplementary Figure 2**. Location of the microsatellite markers used with respect to the *BRCA1* gene.


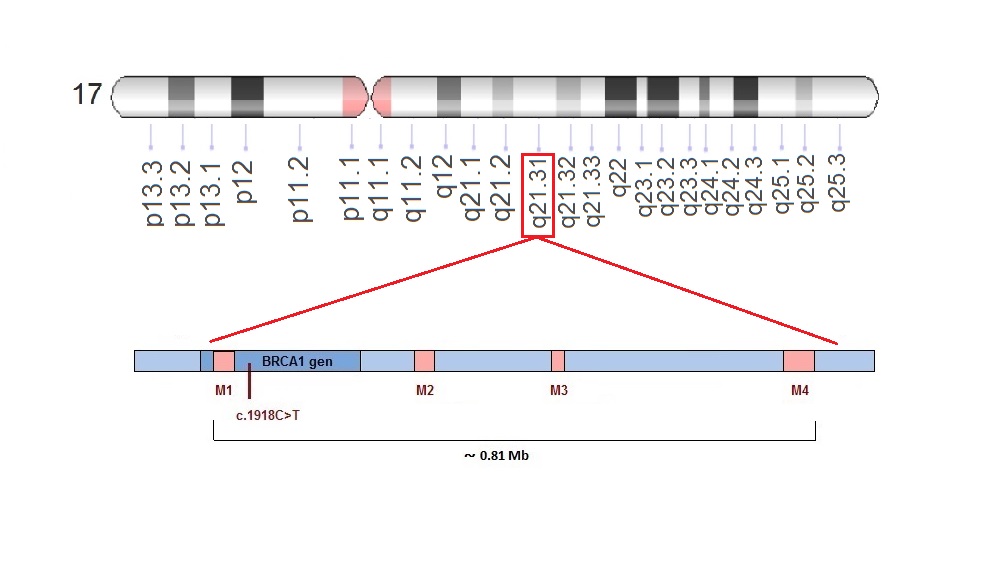


**Supplementary Table 6.** Primer sequences used in the haplotype analysis of the exon 2 deletion in *BRCA2*

(forward primers were labeled with the FAM fluorophore for use in the analysis of fragments). Temp: annealling temperature, bp: base pairs

| **MICROSATELLITE MARKERS IN *BRCA2*** | | | | | |
| --- | --- | --- | --- | --- | --- |
| Marker | | *Forward primer* | *Reverse primer* | Temp (ºC) | Size (bp) |
| M1 | D13S260 | [6FAM] AGATATTGTCTCCGTTCCATGA | CCCAGATATAAGGACCTGGCTA | 57 | 158 - 173 |
| M2 | D13S1493 | [6FAM] ACCTGTTGTATGGCAGCAGT | GGTTGACTCTTTCCCCAACT | 57 | 223 - 248 |
| M3 | D13S153 | [6FAM] AGCATTGTTTCATGTTGGTG | CAGCAGTGAAGGTCTAAGCC | 58 | 212 - 236 |

**Supplementary Figure 3.** Location of the microsatellite markers used with respect to the *BRCA2* gene


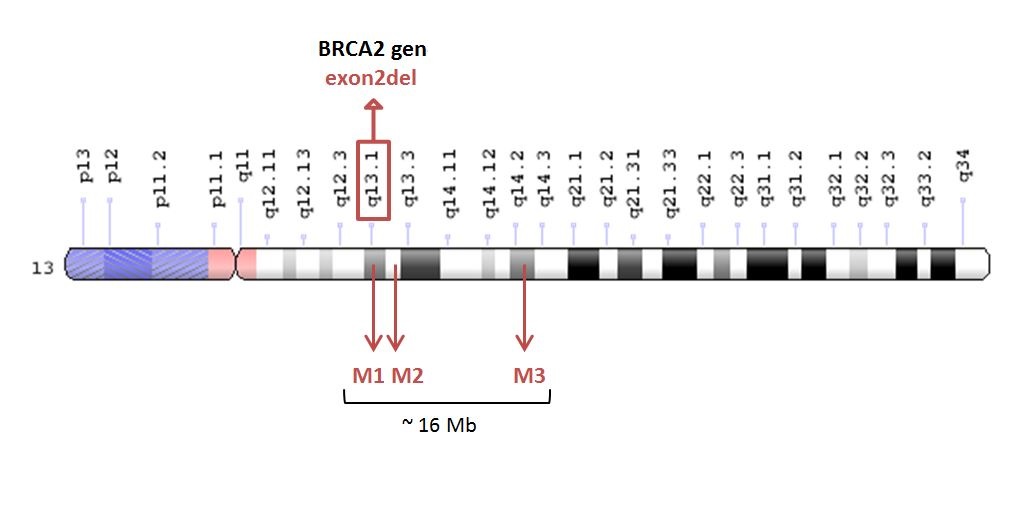


**Supplementary Table 7.** Primer sequences used in the haplotype analysis of the c.8251_8254del variant in *ATM* (forward primers were labeled with the FAM fluorophore for use in the analysis of fragments). Temp: annealling temperature, bp: base pairs

| **MICROSATELLITE MARKERS IN ATM** | | | | | |
| --- | --- | --- | --- | --- | --- |
| Marker | | *Forward primer* | *Reverse primer* | Temp (ºC) | Size (bp) |
| M1 | D11S4078 | [6FAM] AGGCTAACCAGCCAACATT | CGGGCTATAAAACTCAAGTCTC | 61 | 135-159 |
| M2 | D11S1391 | [6FAM] TGCATGCATACATACATACATACA | CATCCATCCCTCTGTCTCTG | 55 | 158-178 |
| M3 | D11S1781 | [6FAM] AGCTGTTCTTGTCACAGGAGAG | ACAAATTGTCAGTGCCCC | 55 | 243-251 |
| M4 | D11S1390 | [6FAM] GGGTGGAATCCTTCAGAATT | AAATATTACCGGGCTTGGAC | 57 | 145-165 |
| M5 | D11S4176 | [6FAM] AGACTCTCTCGTCCTCAGGG | GGGTAGCACTCCCAGGTT | 62 | 224-254 |
| M6 | D11S4197 | [6FAM] TGAGGTCAATGTTGGTTTC | TAGTAGAATCTCATAGGTTCTGTGG | 57 | 227-271 |

**Supplementary Figure 4.** Location of the microsatellite markers used with respect to the *ATM* gene


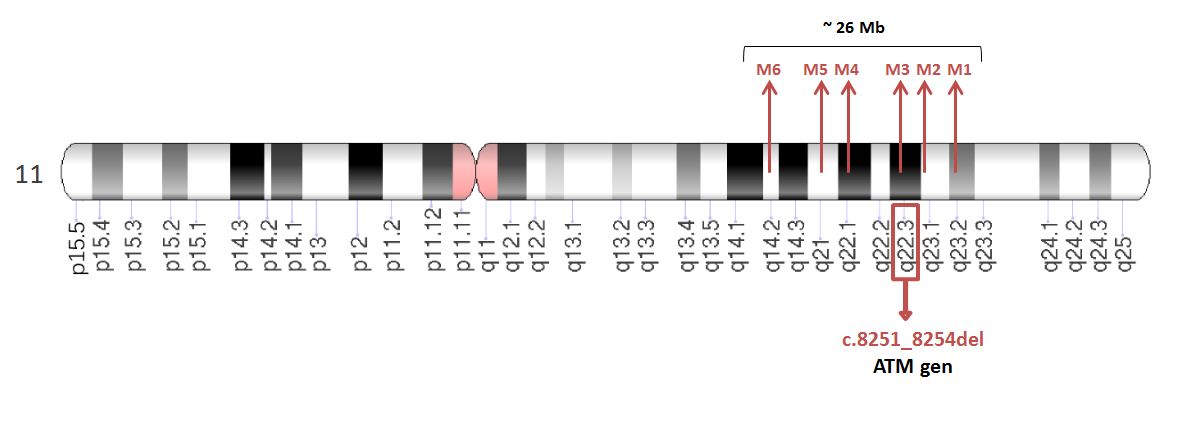


**Supplementary Table 8.** Haplotypes obtained in carriers of the c.1918C>T variant in *BRCA1.* (the common haplotype is indicated in green) IC: index case; M: microsatellite

|  | M1 (D17S855) | | M2 (17-3858) | | M3 (D17S1326) | | M4 (17-3930) | |
| --- | --- | --- | --- | --- | --- | --- | --- | --- |
| CI 1 | 139 | 144 | 122 | 126 | 89 | 108 | 308 | 298 |
| CI 2 | 139 | 146 | 122 | 126 | 89 | 106 | 308 | 298 |
| CI 3 | 139 | 146 | 122 | 114 | 89 | 102 | 308 | 301 |
| CI 4 | 139 | 144 | 122 | 128 | 89 | 106 | 308 | 298 |
| CI 5 | 139 | 148 | 122 | 134 | 89 | 106 | 308 | 286 |
| CI 6 | 139 | 146 | 122 | 128 | 89 | 106 | 301 | 298 |
| CI 7 | 139 | 146 | 122 | 118 | 89 | 89 | 308 | 289 |
| CI 8 | 139 | 144 | 122 | 120 | 89 | 89 | 308 | 295 |

**Supplementary Table 9.** Haplotypes obtained in carriers of the exon 2 deletion in *BRCA2.* (the common haplotype is indicated in green) IC: index case; M: microsatellite

|  | M1 (D13S260) | | M2 (D13S1493) | | M3 (D13S153) | |
| --- | --- | --- | --- | --- | --- | --- |
| CI 1 | 161 | 167 | 223 | 227 | 223 | 219 |
| CI 2 | 161 | 160 | 223 | 235 | 226 | 226 |
| CI 3 | 161 | 167 | 227 | 231 | 221 | 228 |
| CI 4 | 161 | 161 | 223 | 227 | 223 | 219 |
| CI 5 | 161 | 165 | 223 | 223 | 223 | 230 |
| CI 6 | 161 | 163 | 223 | 223 | 211 | 219 |
| CI 7 | 161 | 163 | 223 | 227 | 211 | 217 |
| CI 8 | 161 | 163 | 223 | 227 | 223 | 219 |
| CI 9 | 161 | 163 | 223 | 235 | 223 | 211 |
| CI 10 | 161 | 157 | 223 | 223 | 211 | 219 |
| CI 11 | 161 | 163 | 227 | 227 | 215 | 228 |
| CI 12 | 161 | 157 | 223 | 235 | 215 | 219 |

**Supplementary Figure 5.** Haplotypes of carriers of the c.8251_8254del variant in *ATM.* (The common haplotype is shown in green) BC: breast cancer, bBC: bilateral breast cancer, PC: pancreatic cancer, PrC: prostate cancer, TC: thyroid cancer, MTC: mantle cell lymphoma, Mel: melanoma


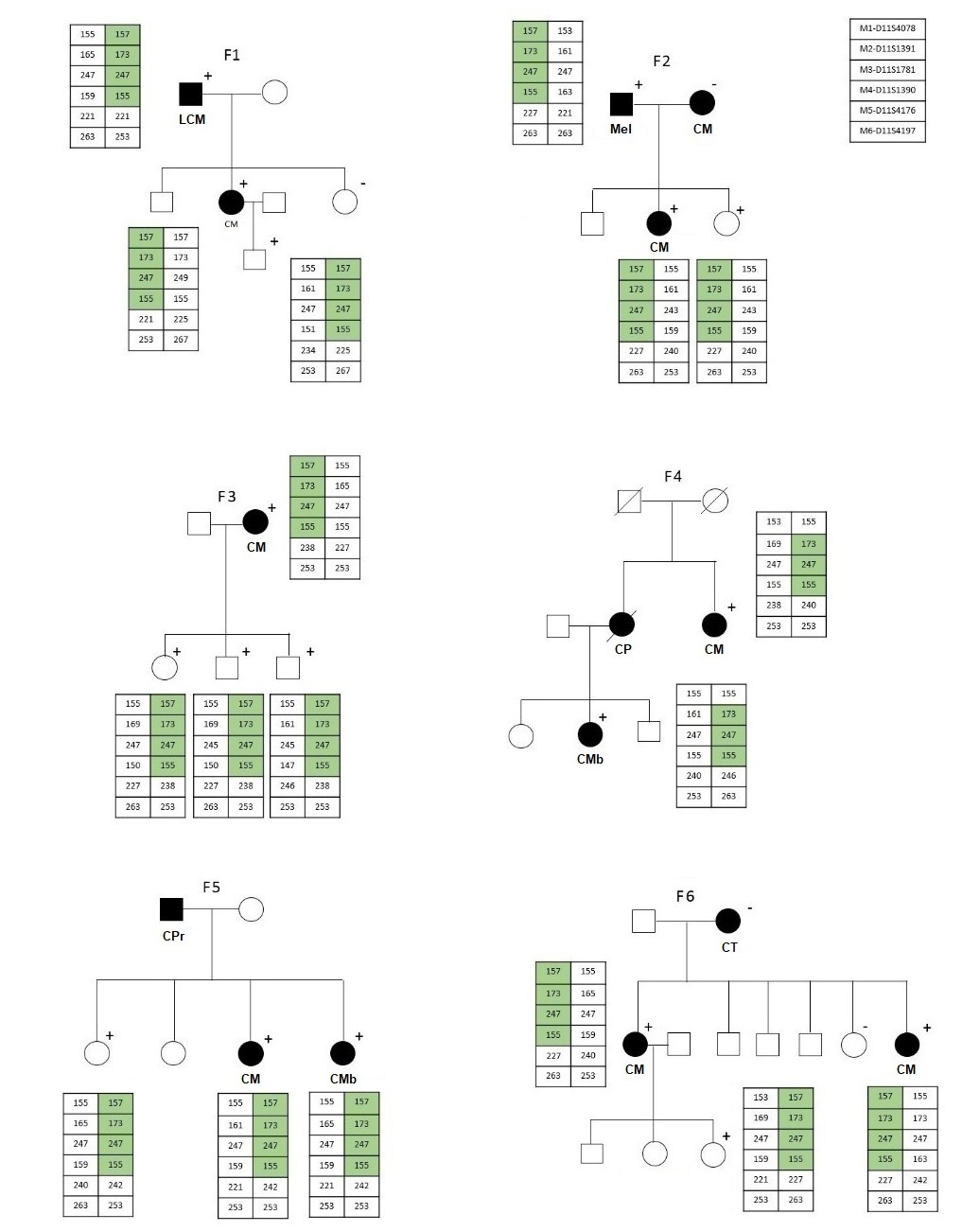

Supplement: Supplementary file 1 — Supplementary Material [file j_almed-2023-0103_suppl_001.doc]
